# Re-evaluation of solutions to the problem of unprofessionalism in peer review

**DOI:** 10.1186/s41073-020-00107-x

**Published:** 2021-02-16

**Authors:** Travis G. Gerwing, Alyssa M. Allen Gerwing, Chi-Yeung Choi, Stephanie Avery-Gomm, Jeff C. Clements, Joshua A. Rash

**Affiliations:** 1 Department of Biology, University of Victoria, Victoria, British Columbia Canada; 2 Sidney Museum and Archives, Sidney, British Columbia Canada; 3 School of Environmental Science and Engineering, Southern University of Science and Technology, Shenzhen, China; 4 Environment and Climate Change Canada, National Wildlife Research Center, Ottawa, ON Canada; 5 Aquaculture and Coastal Ecosystems, Fisheries and Oceans Canada Gulf Region, Moncton, New Brunswick Canada; 6 Department of Psychology, Memorial University of Newfoundland, St. John’s, Newfoundland Canada

**Keywords:** Mental health, Peer review, Toxic culture

## Abstract

Our recent paper (10.1186/s41073-020-00096-x) reported that 43% of reviewer comment sets (*n*=1491) shared with authors contained at least one unprofessional comment or an incomplete, inaccurate of unsubstantiated critique (IIUC). Publication of this work sparked an online (i.e., Twitter, Instagram, Facebook, and Reddit) conversation surrounding professionalism in peer review. We collected and analyzed these social media comments as they offered real-time responses to our work and provided insight into the views held by commenters and potential peer-reviewers that would be difficult to quantify using existing empirical tools (96 comments from July 24th to September 3rd, 2020). Overall, 75% of comments were positive, of which 59% were supportive and 16% shared similar personal experiences. However, a subset of negative comments emerged (22% of comments were negative and 6% were an unsubstantiated critique of the methodology), that provided potential insight into the reasons underlying unprofessional comments were made during the peer-review process. These comments were classified into three main themes: (1) forced niceness will adversely impact the peer-review process and allow for publication of poor-quality science (5% of online comments); (2) dismissing comments as not offensive to another person because they were not deemed personally offensive to the reader (6%); and (3) authors brought unprofessional comments upon themselves as they submitted substandard work (5%). Here, we argue against these themes as justifications for directing unprofessional comments towards authors during the peer review process. We argue that it is possible to be both critical and professional, and that no author deserves to be the recipient of demeaning *ad hominem* attacks regardless of supposed provocation. Suggesting otherwise only serves to propagate a toxic culture within peer review. While we previously postulated that establishing a peer-reviewer code of conduct could help improve the peer-review system, we now posit that priority should be given to repairing the negative cultural zeitgeist that exists in peer-review.

## Introduction

In 2020 our team of investigators published two papers that assessed the institution of academic peer-review. The first paper quantified the frequency of unprofessional comments directed towards authors, and the occurrence of incomplete, inaccurate or unsubstantiated critiques within reviewer comment sets [1]. The second paper explored the establishment of a reviewer code of conduct as one proposed solution [2]. These manuscripts highlighted ways the focus of the peer-review process can shift from the scientific rigour of the submitted work to the personal characteristics of the authors, thus becoming harmful, especially to early career investigators and underrepresented groups [3–5]. We posited that no systemic change to peer-review – such as the adoption of different peer review models – will improve this system if those who act within it do not uphold the ideals of professional evaluation of submitted manuscripts [1, 2].

While reading 1491 sets of peer-reviewer comments provided powerful insights into the peer review process, it was the subsequent feedback that we received through social media after publishing these manuscripts that was truly profound. Such comments represent real-time responses to our work and provide insight into the views held by commenters and potential peer-reviewers that would be difficult to quantify using existing empirical tools. It is thus useful to digest this online feedback to further strive toward increasing professional conduct in peer review. As such, the purpose of this manuscript was to quantify the relative prevalence of positive and negative comments received over social media about our work between July 24th (date of first publication) and September 3rd, 2020, to better understand the factors that underlie unprofessional comments in peer review.

## Methods

We retrieved all comments pertaining to our previous manuscript that were posted on social media (i.e., Twitter, Instagram, Facebook, and Reddit) between July 24th and September 3rd, 2020. The comments were collected using the Altmetric summary accessed via Publons (https://www.altmetric.com/details/86395746?src=bookmarklet#score). Promotional posts made by the authors and by the journal in which our study was published were not considered, resulting in a total of 96 assessed comments. All text within a single post was extracted without identifying information in an attempt to remove potential for bias, and then categorized as positive (praised or agreed with the article), negative (criticized or disagreed with the article), or not of relevance (commented on a subject unrelated to the article). As such, a single post could contain more than one comment that pertained to more than one category (e.g., a single post could contain information that was positive as well as negative). Comments within each category were sub-categorized based on inductive reasoning. Text was anonymized by JAR and coding performed by TGG. Codes were reviewed for accuracy and no disagreements were noted.

### Limitations

Several limitations must be considered when interpreting the public comments that we collated. First, comments were obtained from social media posts. It is unclear whether these commentators regularly engage in the peer-review process, or whether they would generalize to views held by the peer-review community. Second, social media is dynamic, and posts can be deleted or edited. Third, the comments that we collated may be subject to selection bias. Individuals who are prone to share their opinions on social media are likely those who are most passionate about the issue of peer-review. Finally, social media comments were coded by the intended targets of such comments, and therefore may be biased. All comments are freely available online and can be assessed independently.

### Ethics approval

Ethical approval was not necessary for this investigation. According the Tri-Council Policy Statement: Ethical Conduct for Research Involving Humans (TCPS 2; 2014), research which relies exclusively on publicly available information does not require ethics review if the information is legally accessible to the public and appropriately protected by law, and/or there is no reasonable expectation of privacy.

## Results and discussion

Table 1 depicts feedback received from social media activity that occurred between July 24th and September 3rd. Our previous manuscript received strong attention during this timeframe, being shared 221 times. This work stimulated substantial discussion, eliciting 96 assessable comments over social media within the six-week period. As of September 7th, 2020, the paper had an Altmetric score of 206, including 296 tweeters, 3 blogs, 1 Facebook page, and 1 share on Reddit. At the time, this paper generated the most Altmetric feedback of any article published in Research Integrity and Peer Review and was considered among the top 5% of all research outputs measured by Altmetric (https://www.altmetric.com/details/86395746?src=bookmarklet#score).

**Table 1:** Coding of comments obtained from social media

	**Total**	**Facebook**	**Twitter**	**Blogs**	**Reddit**
**Comment Received**	*N*=96	*N*=18	*N*=64	*N*=8	*N*=6
***Positive Comments***					
Praise of study	57 (59%)	6 (30%)	45 (70%)	1 (13%)	5 (83%)
Sharing personal experience	15 (16%)	1 (5%)	14 (22%)	0 (0%)	0 (0%)
***Neegative Comments***					
Unsubstantiated critique of study methodology	6 (6%)	0 (0%)	2 (3%)	4 (50%)	0 (0%)
Forced niceness will weaken peer-review	5 (5%)	2 (10%)	2 (3%)	1 (13%)	0 (0%)
Difficulty adopting perspective of others	6 (6%)	3 (15%)	2 (3%)	1 (13%)	0 (0%)
Authors brought unprofessional comments upon themselves by submitting substandard work	5 (5%)	4 (22%)	1 (1%)	0 (0%)	0 (0%)
***Not of relevance***	2 (2%)	0 (0%)	0 (0%)	1 (13%)	1 (17%)
**Sharing over Social Media**	**Total**	**Facebook**	**Twitter**	**Blogs**	**Reddit**
Tweets / Retweets / Shared without comments	221	5	215	1	0

Note. Some feedback sources contained more than one category of reviewer feedback, resulting in some columns which total more than 100%

### Positive comments

Overall, 72 (75%) comments were positive, of which 57 (59%) are supportive to the publication (i.e., commended the study, expressed shock at the examples, and supported attempts to improve the current peer review process), and 15 (16%) shared similar personal experiences (Table 1; “be prepared to [feel] despair at the unprofessional comments they quote … no surprises but still”). These commenters shared personal and painful stories of unprofessional comments from a reviewer and agreed that such behaviour must be remedied.

### Negative comments

A subset of commenters took umbrage with our work, and the subject of this disagreement has proven insightful. Overall, 22 (22%) comments were categorized as negative, with 6% of all comments criticizing the study methodology of our previous manuscript (e.g. “these methods are really really bad,” “this is a crappy study”) and validity of observed results (Table 1), without highlighting shortcomings of the manuscript or offering constructive ways to improve.

The rest of the observed negative comments were classified as belonging to 3 general themes: 1) forced niceness will adversely impact the peer-review process and allow for publication of poor-quality science (5% of comments); 2) dismissing comments as not offensive to another person because they were not deemed personally offensive to the reader (6%); and 3) authors brought unprofessional comments upon themselves by submitting substandard work (5%). We explore these sub-categories of comments below and expand upon why such beliefs are harmful to the peer-review process. Our goal is not to attack specific individuals, so exact comments will not be provided. Rather, our motivation is to draw attention to the negative cultural zeitgeist that exists in peer review, in a hope that discussion will stimulate improvement.

### Forced niceness will adversely impact the peer-review process

Comments in this category raised the point that forced niceness and civility within peer-review would impair a reviewer’s ability to convey a critical evaluation of submitted manuscripts. Commenters detailed how this would result in a watering down of the literature due to an increase in the publication of substandard work. This is an argument that we disagree with on principle, as it seems perfectly reasonable that a reviewer could be both professional and critically reject substandard work. Fortunately, we have data to support this postulate. Our previous manuscript evaluated 1491 reviewer comment sets and scored the number of comment sets that included unprofessional comments (comments that focused on an author’s sex, gender, age, race, place of origin, or native language, as well as comments that could be interpreted as insulting or demeaning), as well as the proportion of comment sets that included inaccurate, incomplete, or unsubstantiated critiques (IIUC). This allowed us to quantify the proportion of reviewer comment sets that were unprofessional as well as a substandard critique of the submitted manuscript. We observed that 43% (641) of comment sets included at least one unprofessional comment or IIUC. Put another way, 88% of comment sets did not contain unprofessional elements, 59% contained no IIUCs, and 57% contained neither unprofessional comments nor IIUCs. As such, a majority of reviewer comment sets were likely both professional and critical; pointing out flaws or failings in the submitted work, suggesting major revisions or outright rejection, without demeaning the author/work or commenting upon race, gender, sex, place of origin, or native language. Therefore, it is empirically possible for reviewers to be professional, while also critically examining a submitted manuscript. In fact, it seems that most reviewers are doing just that. Bluntly put, “forced niceness” does not appear to constitute a valid argument in defence of unprofessional comments in peer review.

### Challenges with perspective taking: I was not offended, and neither should you

This group of commenters took exception to some of the examples of unprofessional comments provided in Gerwing et al. [1]. Common points of friction were reviewers that commented on the language of origin of the author (“English is clearly not your first language”) or the application of negative adjectives (“the writing is truly, truly awful”). Commenters noted that such a comment would not have offended them and/or recipients who were offended were being overly sensitive. We find this argument unconvincing as communication is bidirectional, and the recipient must be considered. For instance, it is the responsibility of the author of a scientific paper to ensure their findings are communicated as clearly and professionally as possible. This responsibility is no different for the author of a peer review comment (i.e., the reviewer). Scientists are a diverse group, and reviewers must consider this when writing comments. It is incumbent upon peer-reviewers to consider the perspective of their scientific colleague and contemplate how a colleague with different life experiences would receive and interpret a comment. In our view, this is not forced niceness, nor will such consideration contribute to the watering down of the scientific literature. Considering the recipient of your feedback is merely the maintenance of professional standards of communication. While genuine misunderstandings will be impossible to avoid, comments on certain subjects will always be rife with the potential for harm. Specifically, comments that focus on the sex, gender, age, race, place of origin, and native language of the author, or comments that emphatically utilize negative adjectives to describe the manuscript are very likely to offend. More practically, such comments can be counter-productive, obscuring a potentially constructive point.

### Authors brought unprofessional comments upon themselves as they submitted a subpar manuscript

These comments were perhaps the most troubling to read. Several commenters suggested that poor-quality manuscripts should not be submitted for peer-review and that unprofessional reviewer comments were a justified product of being forced to review submissions or resubmissions that were perceived to be of poor quality. These comments often centered around a perception that command of the English language was problematic. Such comments are troubling in many respects, not least of which is the lack of empathy for scholars forced to publish in a language not of their choosing. It is worth noting that “poor quality” can be a subjective judgement, particularly when such a judgement is made in the absence of a validated tool for the assessment of bias. Indeed, we encountered numerous instances of reviewer discrepancies (e.g., instances where writing was praised by one reviewer while chastised by another) in our previous research [1], that highlights this subjectivity. From an empirical perspective, additional research is needed to determine if in fact lower quality manuscripts or those with poorer readability prompt a higher frequency of unprofessional comments. From a pragmatic stance, it is possible to be critical but polite in pointing out that editorial issues exist. For instance, issues with grammar and writing can be identified without referring to the primary language of the author (e.g. “several instances of grammatical errors were identified and should be addressed in the next version”). Regardless of supposed provocation, no author deserves to be the recipient of demeaning and unprofessional reviewer comments. For academics to suggest otherwise, serves only to promote a toxic culture within peer review.

## Conclusions

After assessing 1491 peer review comment sets, we postulated that establishing a peer-reviewer code of conduct could help remove unprofessional behaviour and IIUCs from the peer-review system. Evaluation of the public response to our previous manuscript has further revealed a troubling and negative cultural zeitgeist that exists within peer review. Social medial comments provide instantaneous and uncensored insights into the views held by commenters, and potential peer-reviewers. Viewpoints that would be difficult to quantify using existing empirical tools. We now posit that a reviewer code of conduct and editorial intervention alone, while a good first step, may be insufficient to stimulate improvement of the peer-review process; at least until the negative cultural zeitgeist that exists in peer-review is repaired. Undoubtably, editors and journals must play a role in mitigating the occurrence of unprofessional comments by providing clear guidance to reviewers about using inclusive and constructive language. However, we argue that a top-down approach will only go so far in addressing this issue, and contend that changing the negative cultural zeitgeist within peer review will also require change from the bottom up, starting with peer reviewers. It is important to point out that even a small number of bad actors can result in the majority of researchers receiving unprofessional reviewer comments [5], a phenomenon that contributes to poor mental health in researchers [5–8]. While peer review is a voluntary and often an unrecognized service activity, the onus of change rests upon reviewers. The level of editorial oversight required to filter out such comments would place too high a burden upon editors, many of whom also volunteer in their positions. As such, unprofessional comments will persist within peer-review until reviewers embrace the idea that feedback can be both professional and critical, and until reviewers put more effort into considering the perspective of authors when wording their comments. It is our hope that bringing attention to these comments will stimulate the discussion required to repair this negative cultural zeitgeist.

Such improvements are far from trivial, but change must happen now. The mental health of underrepresented groups, early career investigators, and researchers in general is poor, and this trend is not improving [6, 9–11]. Peer reviewed publications are the currency of academic career advancement. If peer-review is contributing to deteriorating mental health of researchers, then the process of career advancement could be damaging to our mental health. Therefore, immediate change is required to better protect ourselves and our colleagues.

## Data Availability

Social media comments are freely available online.

## Abbreviations

IIUC: Inaccurate, incomplete, or unsubstantiated critiques
